# Reframing ATR: from the immunologic/nonimmunologic dichotomy to adaptive, innate, and physicochemical drivers within the allergy–inflammation–coagulation axis

**DOI:** 10.3389/fimmu.2026.1892622

**Published:** 2026-09-03

**Authors:** Yoshihiko Sakurai

**Affiliations:** Department of Legal Medicine, Nara Medical University, Kashihara, Nara, Japan

**Keywords:** allergic transfusion reaction, anti-endothelial cell antibodies, coagulation, complement, endotyping, mast cell, thrombo-inflammation

## Abstract

**Background:**

Allergic transfusion reactions (ATRs) remain a significant challenge in transfusion medicine. The traditional binary classification—immunologic versus nonimmunologic, or IgE-mediated versus non-IgE-mediated—fails to capture the full complexity of ATR pathophysiology, as specific allergens are rarely identified.

**Study design and methods:**

This review synthesizes interdisciplinary insights from hemostasis, immunology, and allergy, particularly regarding the pathogenesis of chronic spontaneous urticaria (CSU) to reconstruct the pathophysiology of ATRs and propose a comprehensive new mechanistic framework.

**Results:**

We propose the inflammation–coagulation–allergy (ICA) axis as the primary driver of many ATRs. In this model, the coagulation and complement systems directly activate mast cells via anaphylatoxins and protease-activated receptors (PARs), independent of classical IgE pathways. The components in blood products, particularly biological response modifiers (BRMs) and microparticles (MPs) resulting from storage lesion and potentially anti-endothelial cell antibodies (AECAs) act as triggers. When transfused into a recipient in a physiologically “primed” state due to aging, invasion, or infection, these factors ignite localized thrombo-inflammatory pathways via interaction between coagulation and complement systems, leading to tissue mast cell activation. Consequently, while the basophil activation test (BAT) improves the detection of classical IgE-mediated reactions, it may not capture these coagulation-driven, tissue mast cell-mediated mechanisms.

**Discussion:**

We argue for reframing ATRs not merely as localized cutaneous reactions but as systemic thrombo-inflammatory events. This paradigm shift highlights the potential utility of thrombotic biomarkers (e.g., D-dimer) in endotyping ATRs and points toward novel, mechanism-based therapeutic strategies beyond standard antihistamines.

## 1 Introduction

Adverse reactions to blood transfusions are broadly classified into hemolytic and non-hemolytic reactions. In 2024, the Japanese Red Cross Society reported 26 hemolytic and 3,065 non-hemolytic adverse reactions. Among the latter, allergic transfusion reactions (ATRs) accounted for 2,115 cases, representing over two-thirds of all non-hemolytic reports (1). Fundamentally, an allergy is an immune response to widespread, exogenous substances that induces hypersensitivity. Hypersensitivity is an inappropriate immune response to common, normally harmless antigens, forming a clinical spectrum that ranges from mild (e.g., atopic dermatitis and rhinitis) to severe (e.g., anaphylaxis and anaphylactic shock) (2). The high proportion of ATRs in Japan is likely attributable to the broad capture of mild cases, a relative decrease in febrile non-hemolytic transfusion reactions (FNHTRs) due to universal leukoreduction, and specific classification criteria.

Globally, hemovigilance systems vary in their definitions of allergic reactions, and mild cases are often excluded from routine reporting (3). Consequently, the true incidence of ATRs, including mild cases, remains unclear. In the United States, the reporting of non-severe allergic reactions became voluntary in 2013, obscuring recent prevalence data. Prior to this change, allergic events constituted 46.8% of all reported transfusion reactions, with 92.8% of these classified as non-severe (4). Collectively, these data suggest that ATRs represent a substantial proportion of transfusion-related adverse events worldwide.

Conventionally, allergies have been understood as hypersensitivity reactions driven by one of two immunologic mechanisms: antibody-mediated or cell-mediated (5). Anaphylaxis, the most severe manifestation of allergy, has also seen evolving definitions. Historically divided into “anaphylactic” and “anaphylactoid” reactions, it is now classified as allergic anaphylaxis when mediated by adaptive immune mechanisms (e.g., IgE, IgG, or immune complex/complement activation), and as nonallergic anaphylaxis when triggered by nonimmunologic factors (6).

However, these conventional paradigms struggle to fully explain the pathogenesis of ATRs. In many instances, the causative allergen remains unidentified, and neither IgE nor IgG antibodies are detected. Consequently, current diagnostic testing strategies fail to capture the full clinical spectrum of ATR pathophysiology. As our fundamental understanding of allergy continues to evolve, our perspective on ATRs must adapt accordingly. To address the limitations of the traditional immunologic versus nonimmunologic dichotomy, this review seeks to reconstruct the pathophysiology of ATRs through a novel framework: the inflammation–coagulation–allergy (ICA) axis. By integrating the roles of innate immunity, coagulation, and the complement system, we aim to provide a more comprehensive understanding of these complex transfusion reactions.

## 2 Limitations of the traditional paradigm: adaptive immune mechanisms

Historically, ATRs have been categorized primarily as either Type I hypersensitivity—arising when specific soluble allergens cross-link IgE antibodies and activate mast cells—or Type III hypersensitivity, which involves IgG-mediated immune complexes. However, in the most common clinical manifestations of ATRs, such as pruritus and urticaria, the inciting allergen is frequently unidentified. In such cases, it has been postulated that ATRs develop largely through nonimmunologic mechanisms that enhance the propensity of mast cells to release histamine and other vasoactive mediators. Yet, the precise pathophysiologic pathways underlying this heightened cellular sensitivity have remained elusive. Although the traditional protein-antibody paradigm is the most widely described model for ATRs, it possesses several critical limitations, as outlined below.

### 2.1 IgG-mediated (type III/CARPA)

Within the context of adaptive immunity, well-known examples of ATRs include anaphylaxis driven by anti-IgA antibodies in patients with IgA deficiency (7, 8), as well as reactions induced by other plasma protein-specific antibodies. Many of these are IgG-class antibodies, and the formation of resulting immune complexes has traditionally been thought to trigger complement activation, culminating in anaphylaxis characteristic of a Type III hypersensitivity reaction. Collet et al. described this phenomenon as CARPA (complement activation-related pseudoallergy)—specifically, a complement-dependent, non-IgE-mediated hypersensitivity (9). In the classical IgG-driven CARPA mechanism, anaphylatoxins generated via the complement cascade are presumed to be the primary mediators of the inflammatory and allergic response.

However, growing skepticism has emerged regarding the assumption that anti-IgA antibodies are the definitive cause of allergic transfusion reactions in patients with IgA deficiency (10, 11). Evidence indicates that even when recipients possess pre-existing anti-IgA antibodies, the actual risk of developing a severe ATR remains notably low (12). Consequently, while the IgG-mediated Type III/CARPA mechanism undoubtedly contributes to certain reactions, it is ultimately insufficient to explain the broader pathophysiology of ATRs.

### 2.2 IgG-mediated (FcγR pathway)

In murine models, it has been demonstrated that allergen-bound IgG antibodies can activate neutrophils, monocytes, and macrophages via Fc-gamma receptors (FcγRs). This activation triggers the robust release of platelet-activating factor (PAF), culminating in systemic anaphylaxis (13). Similarly, in humans, these myeloid cells constitutively express the activating receptors FcγRIIA (CD32A) and FcγRIIIA (CD16A). *In vitro* studies have confirmed that stimulation with immune complexes prompts these cells to release PAF, tumor necrosis factor (TNF), interleukin-6 (IL-6), and other inflammatory mediators. Furthermore, circulating PAF levels in humans strongly correlate with the clinical severity of anaphylaxis, providing compelling evidence for the existence of an analogous IgG-mediated FcγR pathway in human pathology (14). While this pathway offers critical insights into the mechanisms of PAF production during an ATR, relying on it in isolation cannot fully account for the diverse clinical spectrum of these reactions.

### 2.3 IgE-mediated (type I)

In general medical discourse, the term “allergy” is most frequently associated with classical IgE-mediated pathways. Within the context of ATRs, IgE-mediated reactions have been documented following the passive transfer of soluble food allergens present in transfused blood components (15, 16). Additionally, instances of passive sensitization have been reported, wherein donor-derived IgE antibodies are transferred to the recipient via transfusion, subsequently triggering an allergic reaction upon the recipient’s exposure to the specific allergen (17, 18). Despite these well-documented phenomena, it is exceedingly rare to definitively detect specific IgE antibodies directed against the intrinsic proteins contained within blood products in patients experiencing an ATR. Consequently, classical IgE-mediated mechanisms are no longer considered the predominant pathophysiologic pathway driving the vast majority of ATRs.

## 3 Innate immunity and coagulation-driven mechanisms

In recent decades, attention has shifted beyond classical adaptive immune mechanisms toward the vital role of innate immunity. The complement system is now recognized as a core component of this response, and growing evidence indicates that innate immunity operates through an integrated immune-inflammatory-coagulation axis, a dynamic network implicated in a wide range of inflammatory and thrombotic diseases (19–21).

In transfusion medicine, the biologic effects of allogeneic blood have long been conceptualized under the framework of transfusion-related immunomodulation (TRIM). TRIM proposes that transfusion can concurrently activate inflammatory and coagulation pathways (22); however, the mechanistic links between these pathways and immediate clinical reactions such as ATRs remain incompletely defined. Integrating the immune-inflammatory-coagulation axis with the TRIM concept provides a pathophysiologic bridge that may better explain ATR phenotypes not accounted for by classical allergen–antibody models.

### 3.1 Lessons from chronic spontaneous urticaria

In ATRs, dermatologic manifestations—particularly urticaria—are frequently observed. Because ATRs involve the direct intravascular introduction of triggers, they often follow a systemic clinical course akin to that of severe food allergies. Drawing upon established insights into the pathogenesis of chronic spontaneous urticaria (CSU) can profoundly deepen our understanding of the urticarial mechanisms underlying ATRs.

In elucidating the pathogenesis of CSU, the direct crosstalk between allergic inflammation and the coagulation cascade has garnered significant attention. Since the initial reports demonstrating elevated markers of coagulation activation in CSU two decades ago (23–25), extensive research has delineated the role of the hemostatic system in driving this disease. Crucially, the involvement of the extrinsic coagulation pathway—initiated by tissue factor (TF) expression on peripheral blood leukocytes and activated vascular endothelial cells—is now well established (24, 26, 27). Furthermore, D-dimer has proven to be a highly reliable thrombotic biomarker that strongly correlates with clinical disease activity (28). Patients with CSU routinely exhibit elevated plasma levels of prothrombin fragment 1 + 2 (F1 + 2), and thrombin generation assays clearly demonstrate an intrinsically enhanced capacity for thrombin production (29).

In CSU, endogenous anticoagulant pathways, such as tissue factor pathway inhibitor (TFPI), remain functionally intact. This prevents a catastrophic “thrombin burst” and protects against overt systemic thrombosis. Nevertheless, the localized hypercoagulable state generates active proteases—including factors XIa, Xa, IXa, thrombin, and plasmin. Historically, these proteases were proposed to directly and effectively cleave complement proteins C3 and C5, acting as an “extrinsic” complement pathway (28, 30, 31). However, the direct cleavage of C5 by highly purified thrombin under strict physiological conditions is currently debated (32, 33). Despite these mechanistic controversies regarding direct enzymatic cleavage, the profound functional cross-talk and amplification loops between the coagulation and complement cascades drive thrombo-inflammation (34).

Through this interplay, the generation of anaphylatoxins (C3a and C5a) potently activates mast cells and basophils (35). This nonimmunologic activation is mediated by specific G-protein-coupled receptors (GPCRs)—namely the anaphylatoxin receptors C3aR and C5aR1, alongside the modulatory C5aR2. Engagement of C3aR and C5aR1 classically signals via Gi/o (and, to some extent, Gq/G16), thereby activating PLCβ and inducing robust intracellular calcium mobilization and subsequent rapid degranulation of vasoactive inflammatory mediators such as histamine (35, 36). Simultaneously, coagulation proteases exert direct cellular effects. The TF/FVIIa complex efficiently generates factor Xa, which stimulates protease-activated receptors 1 and 2 (PAR-1 and PAR-2) (37, 38), while thrombin activates PAR-1, PAR-3, and PAR-4 (39). PARs are unique GPCRs activated through proteolytic cleavage of their extracellular N-terminus, which exposes a tethered ligand that binds intramolecularly to activate the receptor (39, 40). Upon activation, predominantly PAR-2 (and, to a lesser extent, PAR-1) trigger intracellular signaling cascades, primarily via Gq/11 and phospholipase C (PLC) (41, 42). This leads to inositol triphosphate (IP3) generation, intracellular calcium release, and the direct induction of mast cell degranulation. This entire signaling cascade precipitates the release of potent inflammatory mediators, including histamine and tryptase, entirely independent of classical IgE pathways (43, 44).

### 3.2 Coagulation–mast cell cross-talk

The coagulation system can promote mast cell degranulation through the generation of anaphylatoxins and direct activation of PARs, independent of classical allergen–antibody interactions. Thus, activation of the coagulation cascade alone may trigger urticarial symptoms even in the absence of identifiable exogenous allergens or specific antibodies. In CSU, inflammatory stimuli that upregulate endothelial tissue factor (TF) expression drive this localized hypercoagulable state, a phenomenon also observed in acute urticaria (45–47).

**Figure 1:**
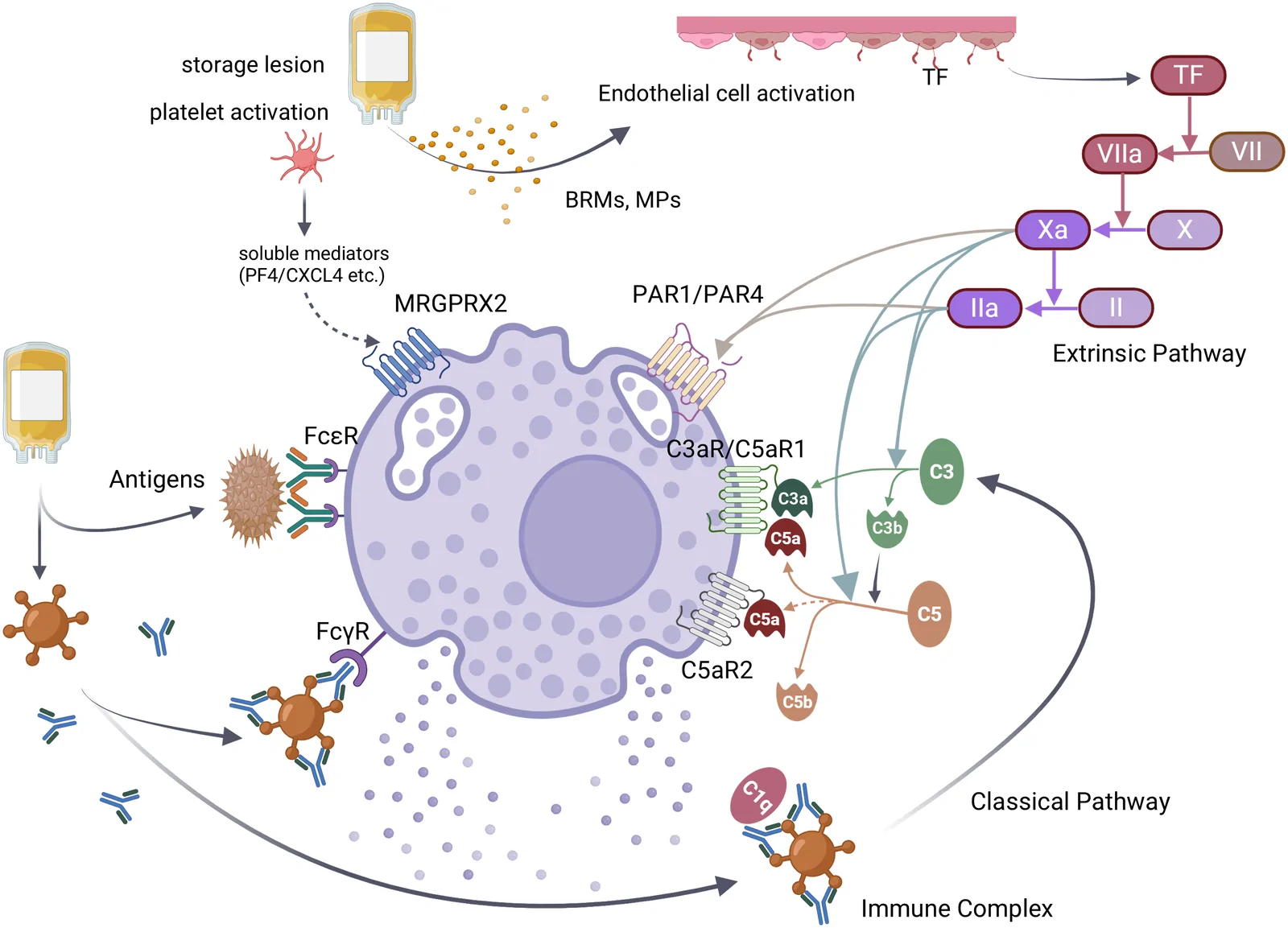
Product-derived triggers and the thrombo-inflammatory activation of mast cells. While classical allergic reactions are driven by IgE-allergen cross-linking and IgG-mediated immune complexes that activate complement, ATRs can also be initiated by distinct non-IgE-mediated pathways. During blood storage, the accumulation of storage lesions (e.g., microparticles [MPs] and biological response modifiers [BRMs]) can trigger the extrinsic coagulation cascade and the complement system. The resulting active proteases (e.g., thrombin and factor Xa) and anaphylatoxins (C3a, C5a) directly stimulate tissue mast cells via protease-activated receptors (PARs) and anaphylatoxin receptors (C3aR, C5aR1, and modulatory C5aR2), independently of specific allergens. Furthermore, soluble mediators released from activated platelets during storage (e.g., PF4/CXCL4) can directly activate the MRGPRX2 receptor. (ATR, allergic transfusion reaction; BRM, biological response modifier; CXCL4, C-X-C motif chemokine ligand 4; MP, microparticle; PAR, protease-activated receptor; PF4, platelet factor 4; TF, tissue factor). This figure was created with BioRender.com.

These findings indicate that allergen exposure is not a prerequisite for urticaria. Rather, coagulation–mast cell cross-talk represents a central non-IgE-mediated pathway and provides a mechanistic framework for ATRs in which no inciting allergen or specific IgE can be identified (**Figure 1**).

## 4 The “seed”: physicochemical and product-derived triggers

As illuminated by the CSU pathophysiologic model, mast cells possess the inherent capacity to degranulate in the complete absence of classical allergens, provided that the coagulation and complement cascades are sufficiently activated. Reflecting this evolving understanding of immune activation, the 2023 revised nomenclature for allergic diseases expanded the traditional Gell and Coombs classification to encompass nine distinct hypersensitivity phenotypes, including direct non-IgE-mediated reactions to chemical agents (Type VII) (48). Consequently, it is imperative to identify the specific, non-allergenic stimuli within blood products that initiate these pathways during a transfusion. This section delineates the principal “seeds”—or product-derived triggers—capable of igniting ATRs, categorizing them into four primary domains: (1) direct physicochemical factors, (2) storage lesion-derived biological response modifiers (BRMs), (3) specific activators of the MRGPRX2 pathway, and (4) donor-derived anti-endothelial cell antibodies (AECAs).

### 4.1 Type VII hypersensitivity: direct physicochemical triggers

A prominent nonimmunologic mechanism of ATRs corresponds to Type VII hypersensitivity (direct, non-IgE-mediated reactions to chemical agents) within the expanded Gell and Coombs classification (48). In various regions, particularly throughout Europe, pathogen reduction technologies for plasma products frequently utilize methylene blue (MB) (49). However, the unintended infusion of residual MB or its photo-oxidized byproducts has been implicated in triggering anaphylaxis in susceptible recipients (50). Mechanistically, MB can act as a hapten, eliciting specific IgE antibody production (51). Furthermore, as a cationic phenothiazine derivative, MB possesses the intrinsic ability to directly induce mast cell degranulation independent of IgE (52). Thus, MB serves as a paradigmatic physicochemical trigger capable of precipitating ATRs via both classical IgE-dependent and entirely IgE-independent pathways.

### 4.2 Storage lesion–derived BRMs

Beyond specific chemical additives, a far more universal set of triggers arises from the cellular components and biological response modifiers (BRMs) that accumulate within blood products during routine storage (collectively known as the “storage lesion”). These endogenous BRMs encompass a spectrum of pro-inflammatory cytokines—including IL-1β, IL-6, IL-8, TNF-α, and MCP-1—as well as extracellular microparticles (MPs) released from degenerating cells. Although universal leukoreduction has significantly mitigated the accumulation of leukocyte-derived BRMs (53), the mechanical stress and cellular adhesion inherent to the leukofiltration process itself can inadvertently activate residual leukocytes, prompting the *de novo* release of cytokines and other vasoactive mediators (54–57). This issue is particularly pronounced in platelet concentrates, where soluble factors released by activated platelets—such as RANTES (CCL5), platelet factor 4 (PF4), and soluble CD40 ligand (sCD40L)—progressively accumulate over the storage period (58). Furthermore, platelet-derived MPs function as potent intercellular messengers that actively promote vascular inflammation (59, 60). During storage, the disruption of cellular membrane phospholipid asymmetry leads to the externalization of phosphatidylserine (PS) on these MPs. This exposed PS provides a highly catalytic scaffold for coagulation factors, endowing the MPs with robust procoagulant activity that potently accelerates the coagulation cascade. Additionally, MPs derived from stored blood components have been shown to directly cleave and activate complement C3 (61, 62). Taken together, these observations suggest that the transfused blood product itself acts not merely as a passive vehicle for allergens, but as a “direct enhancer of coagulation and complement activation,” fully capable of triggering the thrombo-inflammatory pathways that culminate in an ATR.

### 4.3 MRGPRX2-mediated activation

A pivotal recent discovery in allergy and immunology is the identification of the mast cell-specific receptor MRGPRX2. This receptor directly recognizes a broad array of cationic drugs and endogenous basic peptides—including substance P, as well as antimicrobial peptides derived from neutrophils and eosinophils—triggering robust mast cell degranulation in an entirely IgE-independent manner (63, 64). During the routine storage of blood products, cationic proteins such as leukocyte-derived defensins and platelet factor 4 (PF4) are released from disintegrating residual cells and progressively accumulate. Upon transfusion, these concentrated cationic mediators possess the potential to directly stimulate MRGPRX2 on the recipient’s tissue mast cells (65, 66). Consequently, the MRGPRX2 axis represents a crucial, parallel non-IgE/IgG pathway driving ATRs, functioning independently of—yet potentially synergistically with—the coagulation-complement activation cascade.

### 4.4 Anti-endothelial cell antibodies

**Figure 2:**
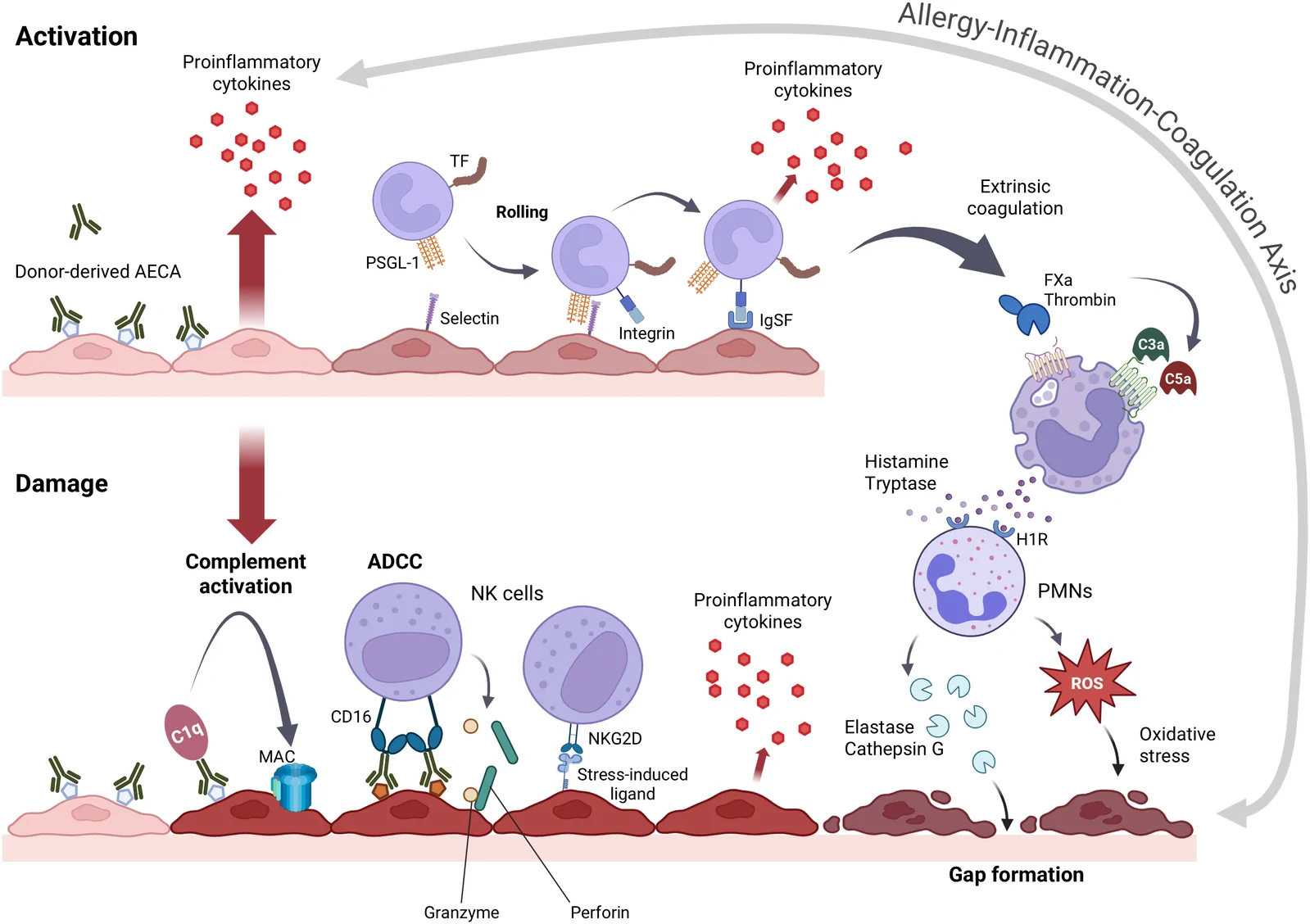
Proposed comprehensive thrombo-inflammatory mechanism driven by anti-endothelial cell antibodies (AECAs). In this hypothesized model, donor-derived AECAs bind to the primed recipient endothelium, igniting a progressive, multifaceted thrombo-inflammatory cascade that evolves from initial cellular activation to severe endothelial damage. Endothelial Activation (Upper panel): AECA binding is proposed to stimulate the initial release of proinflammatory cytokines and induce the expression of adhesion molecules (selectins and IgSF). This would promote the rolling and firm adhesion of circulating leukocytes via PSGL-1 and integrins. These activated leukocytes express tissue factor (TF), initiating the extrinsic coagulation cascade and generating active proteases, including factor Xa (FXa) and thrombin. Endothelial Damage (Lower panel): As the reaction progresses, the endothelium is hypothesized to sustain profound structural and cellular damage. AECAs trigger the classical complement system (via C1q), leading to the generation of anaphylatoxins (C3a, C5a) and the deposition of the membrane attack complex (MAC). Concurrently, natural killer (NK) cells recognize AECA-coated endothelial cells via CD16 and stress-induced ligands (via NKG2D), executing antibody-dependent cellular cytotoxicity (ADCC) through the release of perforin and granzyme. This exacerbates direct endothelial injury and further amplifies proinflammatory cytokine release. Effector Phase & Gap Formation: The converged signals from coagulation proteases (FXa, thrombin) and anaphylatoxins (C3a, C5a) forcefully stimulate tissue mast cells. The subsequent massive release of histamine and tryptase activates polymorphonuclear neutrophils (PMNs) via histamine H1 receptors (H1R). These activated PMNs release reactive oxygen species (ROS), elastase, and cathepsin G, culminating in severe oxidative stress and structural gap formation (vascular leakage). Ultimately, this proposed positive feedback loop—driven by the allergy-inflammation-coagulation axis—could result in the robust systemic reactions characteristic of severe ATRs. (ADCC, antibody-dependent cellular cytotoxicity; AECA, anti-endothelial cell antibody; IgSF, immunoglobulin superfamily; MAC, membrane attack complex; NK cell, natural killer cell; PMN, polymorphonuclear neutrophil; PSGL-1, P-selectin glycoprotein ligand-1; ROS, reactive oxygen species; TF, tissue factor). This figure was created with BioRender.com.

The presence of donor-derived antibodies—particularly AECAs—as primary triggers within transfused blood products has likely been profoundly underestimated. Historically, the field has focused heavily on antibodies directed against human leukocyte antigens (HLAs) and human neutrophil antigens (HNAs) as the primary cause behind transfusion-related acute lung injury (TRALI). For example, HNA-3a antibodies specifically target choline transporter-like protein 2 (CTL2) expressed on endothelial cells (67), leading to the direct disruption of the vascular endothelial barrier (68, 69) (**Figure 2**).

AECAs similarly bind to and activate vascular endothelial cells, inducing robust TF expression that initiates the extrinsic coagulation cascade and establishes a localized procoagulant state. As this thrombo-inflammatory cascade generates potent anaphylatoxins and thrombin—which subsequently activate mast cells—the transfusion of AECA-containing products may precipitate not only TRALI but also systemic urticaria and frank ATRs. Indeed, AECAs are well-documented drivers of vascular inflammation in the pathophysiology of vasculitis (70), and they possess the distinct capacity to trigger the critical linkage between inflammation and coagulation (a pathophysiologic state we have previously conceptualized as “autoimmunothrombosis” in the context of Kawasaki disease) (71). Furthermore, given that autoantibody titers universally tend to increase with advancing age due to immunosenescence (72),the rigorous investigation of donor-derived AECAs—especially those originating from an increasingly aging blood donor demographic (73)—is highly warranted.

### 4.5 Product-specific risk gradient and the impact of leukoreduction filters

Given the profound impact of storage lesions, platelet concentrates inherently pose the highest risk for eliciting ATRs. Platelet-derived mediators, such as PAF, act as potent stimulators of the MRGPRX2 receptor (74). Furthermore, activated platelets actively release damage-associated molecular patterns (DAMPs) and expose membrane surfaces that trigger both the classical and alternative complement pathways (75). In recent years, the widespread implementation of platelet additive solutions (PAS) has successfully mitigated the incidence of ATRs, primarily by diluting offending plasma-derived risk factors and potentially reducing the baseline activation state of the stored platelets (76, 77).

In the context of massive hemorrhage, there has been a significant resurgence of interest in whole blood resuscitation; however, robust leukoreduction remains a critical safety mandate. Emerging evidence highlights the superior hemostatic efficacy of cold-stored whole blood prepared via platelet-sparing leukoreduction filters (78), underscoring the clear clinical benefits of preserving platelets alongside red blood cells. Nevertheless, this approach presents a unique set of challenges: platelet-derived MPs and BRMs inevitably persist in these specific products, potentially serving as concentrated triggers for both the MRGPRX2 and procoagulant pathways. As the allowable storage duration for these products lengthens, the cumulative burden of storage lesions—and consequently, the risk of initiating an ATR—may correspondingly escalate (79). Viewed through the lens of the ICA axis, these dynamic, product-specific risk gradients necessitate a fundamental paradigm shift in how we approach the biological quality control and safety profiling of blood components.

## 5 The “soil”: recipient factors and the two-hit theory

### 5.1 Priming by invasion and inflammation

A critical clinical observation is that the transfusion of identically stored blood products does not precipitate an ATR in every recipient. This discrepancy highlights the indispensable role of the recipient’s basal biological environment—specifically, their physiological “priming”—which dictates their susceptibility to product-derived triggers. The vast majority of patients requiring transfusion therapy are already subjected to significant physiological stress, such as major surgery, severe trauma, or systemic infection. These invasive insults invariably trigger the systemic release of damage-associated molecular patterns (DAMPs), including high-mobility group box 1 (HMGB1) and extracellular histones (80, 81), which potently activate the innate immune system. Crucially, the vascular endothelium is activated by circulating pro-inflammatory cytokines, leading to the robust surface expression of tissue factor (TF). This state of profound endothelial activation serves as the fertile “soil” required to anchor and amplify subsequent coagulation and complement cascades.

### 5.2 Microinflammation as a chronic priming state

Importantly, physiological priming does not exclusively require an acute, overt systemic insult. Chronic, low-grade inflammation—often termed “microinflammation”—is now recognized as a fundamental driver in the pathogenesis and progression of numerous chronic diseases (82). For instance, a ubiquitous chronic pro-inflammatory state associated with advancing age, known as “inflammaging,” is widely documented (83). Similarly, metabolic syndromes such as obesity perpetuate chronic systemic inflammation (84). Given that the elderly constitute a major demographic of blood transfusion recipients, this baseline low-grade inflammation frequently establishes a chronic “primed state,” rendering their vasculature highly susceptible to the sudden activation of the ICA axis.

### 5.3 The second hit: transfusion

**Figure 3:**
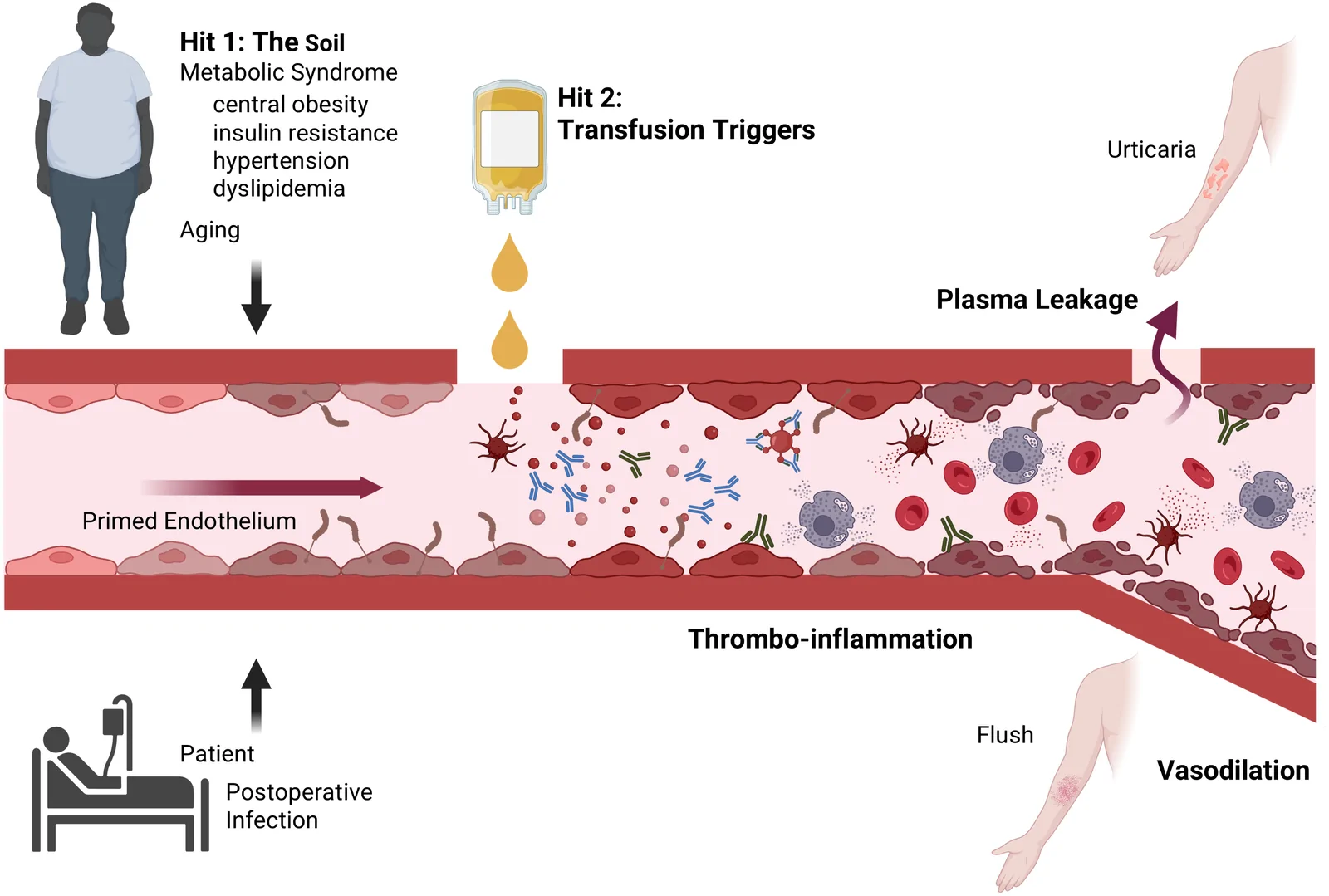
The “Two-Hit” theory of allergic transfusion reactions. The pathogenesis of ATRs is conceptualized as the intersection of a susceptible recipient and a triggering blood product. Hit 1 (The Soil): The recipient’s vascular endothelium is “primed” by acute invasion (e.g., postoperative, infection) or chronic microinflammation (e.g., aging, metabolic syndrome). Hit 2 (Transfusion Triggers): The infusion of product-derived mediators (the “seeds”) onto this primed endothelium ignites a robust thrombo-inflammatory cascade, culminating in systemic mast cell degranulation and clinical manifestations such as vasodilation and plasma leakage. This figure was created with BioRender.com.

When a transfusion is administered to a patient already in this primed state, the donor-derived “seeds”—such as BRMs, MPs, or AECAs—act as a critical “second hit.” While these identical factors might be easily neutralized by the robust homeostatic mechanisms of a healthy individual, in a primed recipient, they ignite an explosive thrombo-inflammatory response. For example, in patients whose vascular endothelial glycocalyx has been compromised by surgical trauma or sepsis (85), product-derived MPs adhere much more readily to the denuded vessel walls, driving the excessive and unchecked progression of immunothrombosis (86). Throughout this process, highly active coagulation proteases, particularly thrombin, directly stimulate perivascular tissue mast cells either through the generation of complement anaphylatoxins or via the direct cleavage of PAR-1 and PAR-4. This culmination of events forces rapid mast cell degranulation, clinically manifesting as acute urticaria and frank ATRs. Ultimately, this framework dictates that ATRs should not be viewed merely as isolated allergic responses to foreign antigens. Rather, they are complex, systemic reactions resulting from the catastrophic disruption of the delicate balance between coagulation and inflammation—occurring when a host, already primed by invasion or chronic disease, receives the additional, precipitating stimulus of a blood transfusion (**Figure 3**).

## 6 Discussion

### 6.1 Diagnostic implications and biomarkers

Recognizing that the pathophysiology of ATRs is fundamentally driven by the ICA axis, a paradigm shift in our diagnostic and testing strategies is imperative. The diagnostic approach to ATRs must be comprehensively restructured into three mechanistic domains: (1) classical IgE-dependent pathways, (2) IgE-independent pathways (e.g., IgG, complement, and MRGPRX2 activation), and (3) coagulation-driven thrombo-inflammatory pathways.

#### 6.1.1 Basophil activation test: pros and cons

BAT provides a functional readout and has enabled identification of previously unrecognized IgE-mediated ATRs (62, 87–89).

However, while BAT demonstrates high diagnostic utility in typical allergic diseases such as food allergies (90), its clinical sensitivity is notably inferior in the context of drug allergies (91). This discrepancy is largely attributed to the fact that many drug hypersensitivities are mediated by IgE-independent mechanisms, such as complement activation via CARPA. Furthermore, in dermatologic manifestations like urticaria—the hallmark symptom of most ATRs—perivascular tissue-resident mast cells are the primary cellular effectors. Consequently, it remains ambiguous whether peripheral basophil reactivity accurately reflects the localized, mast cell-driven mechanisms occurring within the vascular and connective tissues during an ATR.

From a practical standpoint, BAT necessitates specialized flow cytometry equipment and skilled personnel. The passive immune-basophil activation test (pi-BAT)—a recently developed variation in which patient plasma and suspected causative allergens are incubated with basophils from healthy individuals (92)—adds an even higher level of technical complexity, limiting its use in routine clinical screening. Finally, given that approximately 10% to 15% of the general population are intrinsic “non-responders” (individuals whose basophils fail to activate even upon adequate stimulation) (93), it is evident that relying solely on BAT is insufficient to capture the full pathophysiologic spectrum of ATRs.

#### 6.1.2 The usefulness of skin prick test

Compared to the technical complexity of BAT, the skin prick test (SPT) is significantly more straightforward and is widely regarded as the first-line diagnostic approach for classical IgE-mediated allergies (94). In the context of ATRs, utilizing aliquots directly from the blood component provides an accessible screening tool that allows for the direct, *in vivo* observation of localized, IgE-dependent mast cell reactivity (95). Nevertheless, the major limitation of SPT lies in its mechanistic boundaries: it is inherently incapable of detecting IgE-independent pathophysiologic pathways. Specifically, systemic complement activation, MRGPRX2 stimulation by cationic proteins, and coagulation-driven thrombo-inflammatory mechanisms—which we propose as central to the ICA axis—cannot be captured by this modality. Therefore, while SPT remains valuable for ruling in classical Type I reactions, its negativity does not preclude the dynamic, non-IgE-mediated reactions characteristic of most ATRs.

#### 6.1.3 Biomarkers for coagulation-driven ATRs

Conventionally, the measurement of markers of coagulation activation, such as D-dimer, in the setting of a transfusion reaction has been relegated primarily to ruling out severe consumptive coagulopathies like disseminated intravascular coagulation (DIC). However, as extrapolated from the CSU model, hyperactive coagulation proteases directly exacerbate urticarial manifestations by cleaving C3 and C5 into the potent anaphylatoxins C3a and C5a, and by directly stimulating PAR-1 and PAR-2 on tissue mast cells. If this homologous thrombo-inflammatory mechanism operates during an ATR, elevated thrombotic biomarkers—such as D-dimer—may not merely reflect an incidental epiphenomenon, but rather indicate the fundamental “procoagulant spark” driving the allergic response.

Moving forward, it is critical to reinterpret ATRs through the lens of the ICA axis as “systemic inflammatory responses accompanied by dynamic shifts in hemostatic balance.” By actively incorporating the measurement of D-dimer, prothrombin fragment 1 + 2 (F1 + 2), and thrombin-antithrombin (TAT) complexes into the diagnostic algorithm, and by further leveraging rapid point-of-care modalities such as viscoelastic hemostatic assays (VHAs) (96), clinicians may be able to stratify the precise pathophysiology (endotype) of an ATR with unprecedented clinical detail.

### 6.2 Future directions: towards ATR endotyping

The ICA axis framework suggests that many “idiopathic” ATRs arise when product-derived triggers (e.g., BRMs and EVs/MPs) encounter a primed recipient milieu and amplify coagulation–complement signaling, culminating in mast-cell activation via PARs and C5aR1. To translate this concept into precision medicine, four priorities emerge.

- AECA detection: Develop standardized assays to identify donor-derived AECAs with endothelial-activating, TF-inducing potential, especially in cases of recurrent reactions linked to a specific donor/product.
- EV/MP quality metrics: Standardize quantification of EV/MP burden and procoagulant activity as a next-generation quality indicator beyond conventional physicochemical markers.
- Hemostatic biomarkers: Prospectively validate D-dimer, F1 + 2, and TAT (and, where feasible, point-of-care viscoelastic testing) for severity assessment and identification of coagulation-driven phenotypes.
- Mechanism-based endotyping: Move beyond a uniform antihistamine approach by adopting pragmatic endotypes: Type 1 (IgE-mediated; BAT/SPT-positive), Type 2 (IgG/immune complex; complement consumption and/or FcγR-related signatures), Type 3 (MRGPRX2-mediated pseudoallergy), and Type 4 (thrombo-inflammatory/coagulation-driven; elevations in coagulation markers and evidence of coagulation–complement cross-talk, optionally supported by markers of upstream endothelial activation like high-molecular-weight VWF multimers). These endotypes provide a testable roadmap for targeted prevention and therapy.

### 6.3 Conclusion

This review reframes ATRs beyond the traditional “immunologic vs nonimmunologic” or “IgE vs non-IgE” dichotomies by proposing the inflammation–coagulation–allergy (ICA) axis as an integrated framework. In this model, ATRs can be driven not only by classical adaptive immune pathways but also by product-derived triggers (e.g., BRMs and EVs/MPs) that amplify coagulation–complement signaling in susceptible, primed recipients. Downstream effectors such as thrombin, PAR signaling, and anaphylatoxins (e.g., C5a) can activate tissue mast cells and produce urticarial and systemic manifestations even when a specific allergen is not identified.

Clinically, this perspective motivates a mechanism-based diagnostic strategy incorporating IgE-focused testing (BAT/SPT) alongside biomarkers of coagulation activation (e.g., D-dimer, F1 + 2, TAT) to support endotyping and risk stratification. Establishing actionable endotypes may ultimately enable targeted preventive and therapeutic approaches, improving the safety and precision of transfusion medicine.

## Data Availability

The original contributions presented in the study are included in the article/supplementary material.
